# Acute effects of plyometric-based conditioning activity and warm-up music stimuli on physical performance and affective state in male taekwondo athletes

**DOI:** 10.3389/fspor.2023.1335794

**Published:** 2024-01-15

**Authors:** Hamdi Messaoudi, Ibrahim Ouergui, Slaheddine Delleli, Christopher Garrett Ballmann, Luca Paolo Ardigò, Hamdi Chtourou

**Affiliations:** ^1^ High Institute of Sport and Physical Education of Sfax, University of Sfax, Sfax, Tunisia; ^2^ Research Unit: Physical Activity, Sport and Health, National Observatory of Sport, Tunis, Tunisia; ^3^ High Institute of Sport and Physical Education of Kef, University of Jendouba, El Kef, Tunisia; ^4^ Research Unit: Sports Science, Health and Movement, University of Jendouba, El Kef, Tunisia; ^5^ Department of Human Studies, University of Alabama at Birmingham, Birmingham, AL, United States; ^6^ Department of Teacher Education, NLA University College, Oslo, Norway

**Keywords:** ergogenic aids, plyometrics, post-activation performance enhancement, music, combat sports, motivation

## Abstract

**Introduction:**

This study assesses the acute effects of combining a plyometric-based conditioning activity (CA) with different musical stimuli during warm-up on taekwondo (TKD) performance and related psychological aspects.

**Methods:**

In a randomized cross-over study design, 16 male TKD athletes (age: 19.94 ± 1.12 years) performed the TKD-specific agility test (TSAT), frequency speed of kick test (FSKT-10s) and its multiple version (FSKT-mult) under 7 experimental and one control condition. For the experimental conditions, participants experienced varying combinations of music selection process [self-selected (SSM) vs. pre-selected (PSM)], and music tempo [fast (F) vs. slow (S)], within preferred (PG) and non-preferred (NPG) music genre; all in the presence of a plyometric based-CA. Athletes were instructed to rate their perceived exertion (RPE) after each test and their felt arousal (FAS), feeling scale (FS), and motivation after testing completion.

**Results:**

Findings showed that combining a plyometric-based CA with SSMPG at both fast and slow tempo improved TSAT, FSK-10s, FSKT-mult, FAS, motivation, and RPE post-TSAT greater than the other conditions (all *p* < 0.05). Additionally, FSSMPG combined with CA improved FS, and RPE after both FSKT-10s and FSKT-mult better than the other conditions (all *p* < 0.05).

**Conclusion:**

In conclusion, listening to fast self-selected music from a preferred genre in combination with a plyometric-based CA during warm-up setups an individualized warm-up strategy and serves to improve the subsequent specific performances and the related psychological aspects in TKD athletes.

## 1 Introduction

Enhancing sport performance is among the main objectives of athletes, strength and conditioning trainers, and sports scientists. Several strategies have been proposed as training modalities or pre-competition interventions. Post-activation performance enhancement (PAPE) is a warm-up strategy that has been proposed to improve several aspects of athletic performance (1). Specifically, using different conditioning activities (CA), has shown beneficial effects on endurance, explosive, and intermittent exercise performance (2). PAPE represents a unique warm-up modality that is generally considered as a priming of the physiological mechanisms of muscle activation (3–5). These mechanisms include changes in muscle temperature, intramuscular fluid accumulation, and neural activation (6, 7).

PAPE is a phenomenon modulated by different factors, including CA protocol (i.e., mode, duration, effort/pause ratio, rest interval, intensity) and the target population (i.e., sex, training status, age, background) (3–5). In combat sports, the use of CA interventions has revealed mixed results (8, 9). In fact, while some procedures succeed in inducing a PAPE stimulus (8–10), others failed to do so (11). This is likely attributed to the aforementioned factors and the exercise modality (5–7, 12). Even with similar protocols, taekwondo (TKD) investigations have reported inconsistent findings (11, 13), indicating that PAPE is a sensitive phenomenon that will require optimization for specific populations (7, 14). In the context of TKD, plyometrics are among the most used CA which has shown effectiveness at improving sport-specific performances (9, 15). Such modes of intervention resulted in PAPE effects using short rest intervals (15). This was linked to lower fatigue and more pronounced muscle activation induced by plyometric activity (5, 12).

Although integrating a CA during warm-up has been associated with PAPE phenomena in several combat sports circumstances (8–10), application does not always guarantee performance-enhancement (11, 13). To impart PAPE generation, combining a CA with other stimuli has been proposed to induce greater effects than singularly used (16). For instance, low dose of caffeine combined with a plyometric CA during warm-up has been shown to result in synergistic effects for agility and kicking performance in TKD athletes (16). Since affective state and motivation to exercise are key determinants for better sport performance, inducing addition arousal could be a feasible strategy to attenuate psychological stress and physical exhaustion (17). In this regard, music serves as a potent psycho-affective stimulus has been proposed to improve athlete's behavior to exercise (18). For example, listening to music before, during or after exercising improves affective valence, physical performance, perceived exertion, and oxygen consumption (18). From a practical standpoint, listening to music before exercise to impart ergogenic effects is optimal since it is often prohibited during competition (19, 20). Although listening to warm-up music has been repeatedly shown to induce performance enhancement, conflicting results still exist (18). The ergogenic potential of music is dependently linked to the nature of sports activity, inter-individual variability in music preference, and intrinsic components of music (i.e., genre, tempo, intensity) (21–23). In combat sports, evidences regarding potential benefits from listening to warm-up music are limited. In TKD, research has revealed that warm-up music improves performances and reduced fatigue index during specific tasks (24, 25), with greater benefits using preferred than non-preferred music (25). Moreover, tempo of the listened music has been reported to modulate music motivational quality (26). This was confirmed in taekwondo practitioners, where athletes performed specific exercise not only harder but also chose to do so and enjoyed exercising when music was played at a faster tempo (24).

Although CA (9, 10, 15) and warm-up music (24, 25) have been shown independently to impart benefits on TKD performance, whether their combined effects provide further enhancement is unknown. Therefore, this study was designed to investigate the acute combined effects of CA and listening to music during warm-up on the subsequent physical performance and associated psychological responses in TKD athletes. Since physical ability and psychological responses are interrelated and determine each other (27), it was hypothesized that combining a plyometric-based CA with fast self-selected warm-up music would result in greater performances than other conditions.

## 2 Materials and methods

### 2.1 Participants

The sample size was estimated using the G*Power software (Version 3.1.9.7, University of Kiel, Kiel, Germany). Using the F test family (ANOVA: repeated measures, within factors) with α set at 0.05 and power (1-β) set at 0.80, the effect size f calculated was 0.252. The analysis revealed that 14 participants were required to approach an actual power of 82.9%. Sixteen male TKD athletes (Age: 19.94 ± 1.12 years) volunteered to participate in the present study. Athletes were recruited in respect to the following criteria: (i) have a training experience of at least 5 years in TKD, (ii) participate regularly in national competitions for 2 years or more, (iii) have a training routine of 3 days/week at least, and (iiii) did not present any medical restrictions or hearing loss. All athletes were in the pre-competition period. Athletes were asked to follow the same diet, avoid strenuous exercises, and refrain from ergogenic aids 48 h prior to each session. One week before experimentation a meeting with athletes was held, where we explained the protocol, advantages, and disadvantages of the experimentation. The athletes who accepted and volunteered to participate have granted a parental consent and signed a written informed consent. The study was conducted in accordance with the Declarations of Helsinki and approved by a local research ethics committee (CPP SUD N◦ 0332/2021).

### 2.2 Experimental design

**Figure 1:**
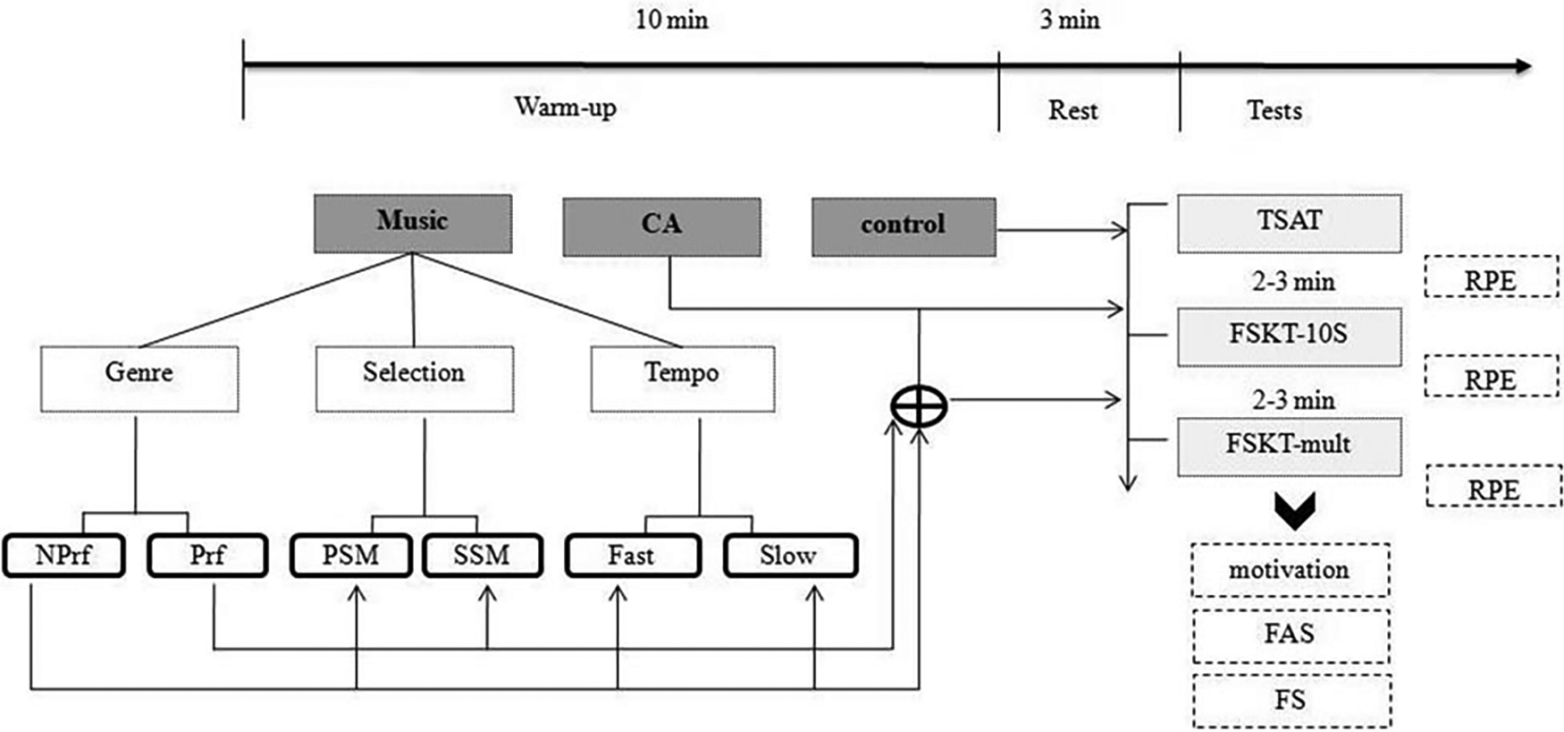
Schematic representation of the study. CA, conditioning activity; NPrf, non preferred; Prf, preferred; PSM, pre-selected music; SSM, self-selected music; TSAT, taekwondo specific agility test; FSKT-10s, frequency speed kick test 10s; FSKT-mult, multiple frequency speed kick test; FAS, felt arousal scale; FS, feeling scale; RPE, ratings of perceived exertion.

In a randomized controlled crossover study design, athletes performed the TKD specific agility test (TSAT) (28), the 10s frequency of speed kick test FSKT-10s (10) and its multiple form (FSKT-mult) (13) and rated their perceived exertion (RPE) (29), feeling scale (FS) (30), arousal (FAS) (31), and motivation (32) after testing session. One week before starting the experimentation, the music selection and familiarization with tests were completed. For the experimental conditions, athletes performed a standardized warm-up (i.e., 10 min of running at moderate intensity) either without a CA or followed by a plyometric-based CA (i.e., 3 sets of consecutive vertical jumps using both legs as fast as possible over an obstacle of 40 cm for 5 s) (9), which has been shown to induce PAPE in combat sports specific tasks (8, 10). Additionally, both warm-up and CA were performed while listening to music or not. After the different warm-up interventions, 3-minute rest intervals between the CA and the subsequent tests were given. This rest interval was used since TKD athletes showed greater sensitivity to stimulus than with longer duration when a plyometric-based CA was applied (15). After each condition, athletes performed, in the same order, the TSAT, the FSKT-10s, and the FSKT-mult with a rest period of 2–3 min between the consecutive tests. After each test, the perceived exertion was rated using the CR-10 scale (29). Moreover, after performing the tests, the FAS, FS, and motivation scores were determined. Since the study was designed to determine the synergetic effect of music and CA, different intrinsic components of music were manipulated including music selection, genre preference, and tempo. The interaction of CA and the manipulated music variables resulted in seven experimental and one control (No CA without music (NM) conditions. Within the preferred music genre (PG), four conditions emerged from the interaction of music selection and tempo as follow: (1) CA with fast (F), self-selected music (SSM) from PG (CA + FSSMPG), (2) CA with slow (S), self-selected music (SSM) from PG (CA + SSSMPG), (3) CA with fast (F), pre-selected music (PSM) from PG (CA + FPSMPG), and (4) CA with slow (S), pre-selected music (PSM) from PG (CA + SPSMPG). Within the non-preferred music genre (NPG), only two conditions were used: (1) CA with fast (F), pre-selected music (PSM) from NPG (CA + FPSMNPG), and (2) CA with slow (S) pre-selected music (PSM) from NPG (CA + SPSMNPG). Moreover, to illustrate the benefits of CA single usage, an additional condition based on CA without music (NM) (i.e., CA + NM) intervention was performed. All these combinations generated a total of 8 conditions. The testing sessions were performed in separate days with 48 h of rest between successive sessions. Moreover, to overcome the effect of diurnal variation of the performance, all sessions were performed at the same time of day (17–19 h). The study design is presented in Figure 1.

#### 2.2.1 Music selection procedure

For the music selection, the subjects were asked to choose their preferred/non-preferred genre of music from a list of 6 genres: rock and roll/hard rock, rap/hip hop, pop, rhythm and blues, country, and dance/electronic. In addition, one of the investigors has selected a list of 10 songs from the Billboard Top 20 of 2021 in that category. The final list of songs was analyzed by the application Decibel: dB sound level meter (developer Vlad Polyanskiy) to check the decibel level of each song. Finally, the subjects choose one preferred song from their favorite genre with a bpm over 120 beats min (i.e., fast self-selected music from the preferred genre) and another preferred song from the same genre with a bpm under 100 beat/min (i.e., slow self-selected music from preferred genre). As well, in the same genre, the investigator chooses randomly a song with a bpm over 120 beat/min (i.e., fast pre-selected music from the preferred genre) and another one under 100 bpm (i.e., slow pre-selected music from the preferred genre). From their non-preferred genre the investigator selected randomly one song with fast rhythm with bpm over 120 beat/min (i.e., fast pre-selected music from non-preferred genre) and another one with a slow rhythm with bpm under 100 beats/min (i.e., slow pre-selected music from the non-preferred genre). Values of bpm during different music conditions were presented in Table 1.

**Table 1:** Musical conditions and tempos.

Music	FSSMPG	FPSMPG	FPSMNPG	SSSMPG	SPSMPG	SPSMNPG
BPM	129 ± 5	130 ± 4	129 ± 4	92 ± 3	94 ± 4	93 ± 5

FSSMPG, fast self-selected music from preferred genre; FPSMPG, fast pre-selected music from preferred genre; FPSMNPG, fast pre-selected music from non-preferred genre; SSSMPG, slow self-selected music from preferred genre; SPSMPG, slow pre-selected music from preferred genre; SPSMNPG, slow pre-selected music from non-preferred genre (NPG); BPM, beat/min.

### 2.3 Testing procedure

#### 2.3.1 TKD specific agility test

**Figure 2:**
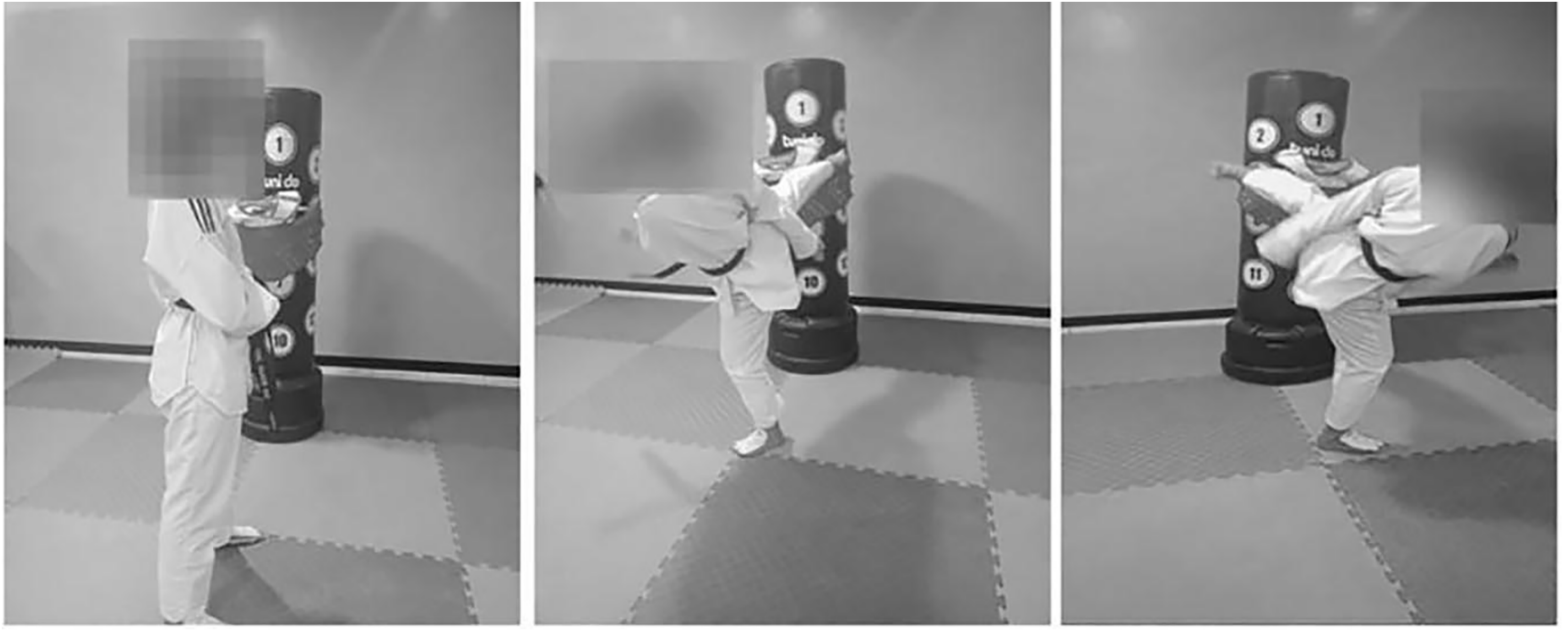
Frequency of speed kick tests’ execution.

From the fighting stance position and behind the start line, the athlete freely advanced to the center mark as fast as possible, and then he turned towards partner 1 using a shift motion and performed a roundhouse kick with their lead leg, turned to the other side, shifted to partner 2, and performed another roundhouse kick with the other lead leg. After that, he returned to the center, moved to partner 3 in the guard position, and performed a double roundhouse kick. Finally, the athlete ran backward to the start/finish line (Figure 2) (28). The kick target was maintained at the trunk height of the tested athlete. The performance time was measured with two sets of single-beam timing lights (Brower Timing Systems, Salt Lake City, UT, USA). Three trials were performed by each athlete, and the best performance was used for the analysis. The intraclass correlation coefficient (ICC) for the test-retest trial (33) for the present study was 0.95.

#### 2.3.2 Ten seconds frequency speed of kick test

**Figure 3:**
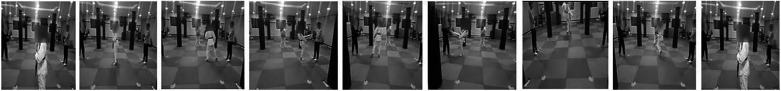
Taekwondo specific agility test set-up.

During the FSKT-10s test, along the 10s bout, each athlete tries to perform the maximum number of kicks (i.e., bandal-chagui) against a hanging bag at the trunk height by alternating the right and left lower limbs (Figure 3). The test's performance index is represented by the total number of kicks (10). The ICC for test-retest trial for the present study was 0.81.

#### 2.3.3 Multiple frequency speed of kick test

Each athlete performed five sets of FSKT-10s with a 10 s of rest interval between repetitions (Figure 3) (13). Performance was determined as the total number of kicks performed in each set and the total number of kicks in the 5 sets, which were subsequently analyzed. The ICC for test-retest trial for the present study was 0.74.

#### 2.3.4 Rating of perceived exertion

Perceived exertion was assessed using the CR-10 Borg scale (29). This is a scale ranging from “0” to “10”, with corresponding verbal expressions, gradually increases with the intensity of perceived sensation (0 = nothing at all; 1 = Very week; 2 = week; 3–4 = Moderate; 5–6 = strong; 7–9 = very strong; and 10 = extremely strong).

#### 2.3.5 Feeling scale

The affective responses were assessed after each test using the feeling scale (30). The FS utilizes a single-item 11-point bipolar rating scale ranging from −5 to +5, with the stem “How do you currently feel?”. Anchors are given at 0 (Neutral) and all odd integers, ranging from “Very Bad” at −5 to “Very Good” at +5.

#### 2.3.6 Felt arousal scale

The Felt arousal scale was used to measure arousal along a 6 point scale ranging from low arousal (1 point) to high arousal (6 points) (31). The participants were instructed to mark the scale based on their perceived activation after each test.

#### 2.3.7 Motivation

The motivation was measured using a 10-point Likert scale that ranged from 0 to 10 with verbal anchors at 10 (extremely motivated), 8 (motivated), 5 (moderate motivated), 2 (weak motivated) and 0 (not motivated) (32).

### 2.4 Statistical analysis

The statistical analysis was performed using SPSS 20.0 statistical software (IBM Corps., Armonk, NY, USA). Data were presented as mean and standard deviation. The Shapiro–Wilk test was used to check and confirm the normality of data sets, and the Levene test was used to verify the homogeneity of variances. Sphericity was tested using the Mauchly test. For TSAT, a one-way analysis of variance (ANOVA) (condition) with repeated measurements was used, with Bonferroni was used as *post hoc* test. Standardized effect size analysis (Cohen's *d*) was used to interpret the magnitude of differences between variables and considered as: trivial (≤0.20); small (≤0.60); moderate (≤1.20); large (≤2.0); very large (≤4.0) (very large); and extremely large (>4.0) (34). For the remaining variables, the non-parametric Friedman test was used with the Wilcoxon signed rank test used as *post hoc*. The correlation coefficient (r) was calculated using the Wilcoxon *z*-scores and the total number of observations (*N*) (i.e., $Z/\sqrt{n}$) and considered as 0.1–<0.3 (small), 0.3–<0.5 (moderate) and ≥0.5 (large) (35). The level of statistical significance was set at *p* ≤ 0.05.

## 3 Results

### 3.1 Physical performance

#### 3.1.1 TSAT

There was a significant impact of conditions (F_7,9_ = 106.94; *p* < 0.001; *η*_p_^2^ = 0.99), with CA + FSSMPG elicited better performance than control (95% CI: −1.55 to −1.08; *p* < 0.001; *d* = −0.61), CA + NM (95% CI: −1.28 to −0.9; *p* < 0.001; *d* = −0.71), CA + SSSMPG (95% CI: −0.83 to −0.28; *p* < 0.001; *d* = −0.24), CA + FPSMPG (95% CI: −1.21 to −0.37; *p* < 0.001; *d* = −0.26), CA + SPSMPG (95% CI: −1.25 to −0.67, *p* < 0.001; *d* = −0.48), CA + FPSMNPG (95% CI: −1.18 to −0.87; *p* < 0.001; *d* = −0.58), and CA + SPSMNPG (95% CI: −1.33 to −0.85; *p* < 0.001; *d* = −0.44). Moreover, CA + SSSMPG elicited lower completion time than control (95% CI: −1.08 to −0.43; *p* < 0.001; *d* = −0.29), CA + NM (95% CI: −0.79 to −0.27; *p* < 0.001; *d* = −0.25), CA + SPSMPG (95% CI: −0.68 to −0.13; *p* = 0.002; *d* = −0.16), CA + FPSMNPG (95% CI: −0.74 to −0.19, *p* < 0.001; *d* = −0.2), and CA + SPSMNPG (95% CI: −0.92 to −0.15; *p* = 0.003; *d* = −0.18). Likewise, CA + FPSMNPG condition elicited lower completion time than the control (95% CI: −0.55 to −0.04; *p* = 0.017; *d* = 0.13) (Table 2).

**Table 2:** Effects of warm-up music and conditioning activity on taekwondo specific agility test (TSAT), frequency speed kick test 10s (FSKT 10s) and multiple frequency speed kick test (FSKT-mult).

Test	Control	CA + NM	CA + FSSMPG	CA + SSSMPG	CA + FPSMPG	CA + SPSMPG	CA + FPSMNPG	CA + SPSMNPG
TSAT(s): M (SD)	6.36 (0.25)	6.13 (0.13)	5.04 (0.17)^a^^,^^c^^,^^d^^,^^e^^,^^g^^,^^ɸ^^,^*	5.6 (0.27)^a^^,^^c^^,^^k^^,^^ɸ^^,^^m^	5.83 (0.4)^b^	6 (0.22)	6.06 (0.18)	6.13 (0.31)
FSKT 10s (*n*): Med/IQR	20/1	21/1	25/1^a^^,^^c^^,^^j^^,^^f^^,^^g^^,^^ɸ^^,^*	23/2^b^^,^^c^^,^^i^^,^^m^	23/2^b^^,^^c^^,^^i^^,^^m^	22/1^b^^,^^i^^,^^m^	21/1	21/1
FSKT mult (*n*): Med/IQR	95.5/4.5	99/5	118/2.75 ^a^^,^^c^^,^^d^^,^^e^^,^^g^^,^^ɸ^^,^*	104/5^b^^,^^β^^,^^i^^,^^m^	103.5/2.5^a^^,^^c^^,^^i^^,^*	104/4.75^b^^,^^β^^,^^m^^,^^i^	101.5/4.75^b^^,^^β^	99/4.75^b^

M (SD), values as Mean (Standard Deviation), Med/IQR, values as Median/Interquartile; CA + NM, Conditioning activity without music (NM); CA + FSSMPG, conditioning activity with fast (F), self-selected music (SSM) from preferred genre (PG), CA + SSSMPG, CA with slow (S), self-selected music (SSM) from preferred genre (PG); CA + FPSMPG, conditioning activity with fast (F), pre-selected music (PSM) from preferred genre (PG); CA + SPSMPG, conditioning activity with slow (S), pre-selected music (PSM) from preferred genre (PG); CA + FPSMNPG, conditioning activity with fast (F), pre-selected music (PSM) from non-preferred genre (NPG); CA + SPSMNPG, conditioning activity with slow (S) pre-selected music (PSM) from non-preferred genre (NPG); s, second; *n*, number.

^a^ Different from control at *p* < 0.001.

^b^ Different from control at *p* < 0.05.

^c^ Different from CA + NM at *p* < 0.001.

^β^ Different from CA + NM at *p* < 0.05.

^d^ Different from CA + SSSMPG at *p* < 0.001.

^j^ Different from CA + SSSMPG at *p* < 0.05.

^e^ Different from CA + FPSMPG at *p* < 0.001.

^f^ Different from CA + FPSMPG at *p* < 0.05.

^g^ Different from CA + SPSMPG at *p* < 0.001.

^k^ Different from CA + SPSMPG at *p* < 0.05.

^ɸ^ Different from CA + FPSMNPG at *p* < 0.001.

^i^ Different from CA + FPSMNPG at *p* < 0.05.

*Different from CA + SPSMNPG at *p* < 0.001.

^m^ Different from CA + SPSMNPG at *p* < 0.05.

#### 3.1.2 FSKT-10s

There was a significant effect of condition (Chi^2^ = 84.417; *p* < 0.001; *df* = 7; *N* = 16), with lower performance in control condition compared to CA + FSSMPG (*z* = −3.54; *p* < 0.001; *r* = 0.89), CA + SSSMPG (*z* = −3.32; *p* = 0.001; *r* = 0.83), CA + FPSMPG (*z* = −3.32; *p* = 0.001; *r* = 0.83), CA + SPSMPG (*z* = −3.23; *p* = 0.001; *r* = 0.81). Also, scores were lower in CA + NM condition compared to CA + FSSMPG (*z* = −3.56; *p* < 0.001; *r* = 0.89), CA + SSSMPG (*z* = −3.54; *p* < 0.001; *r* = 0.89), CA + FPSMPG (*z* = −3.54; *p* < 0.001; *r* = 0.89). The scores were higher in CA + FSSMPG condition compared to CA + SSSMPG (*z* = −2.98; *p* = 0.003; *r* = 0.75), CA + FPSMPG (*z* = −2.79; *p* = 0.005; *r* = 0.70), CA + SPSMPG (*z* = −3.56; *p* < 0.001; *r* = 0.89), CA + FPSMNPG (*z* = −3.54; *p* < 0.001; *r* = 0.89), and CA + SPSMNPG (*z* = −3.56; *p* < 0.001; *r* = 0.89). The scores were higher in CA + SSSMPG condition compared to CA + FPSMNPG (*z* = −3.44; *p* = 0.001; *r* = 0.86), CA + SPSMNPG (*z* = −3.45; *p* = 0.001; *r* = 0.86). The scores were higher in CA + FPSMPG condition compared to CA + FPSMNPG (*z* = −3.33; *p* = 0.001; *r* = 0.83) and CA + SPSMNPG (*z* = −3.32; *p* = 0.001; *r* = 0.83). Higher values were recorded in CA + SPSMPG condition compared to CA + FPSMNPG (*z* = −3.16; *p* = 0.002; *r* = 0.79) and CA + SPSMNPG (*z* = −3.27; *p* = 0.001; *r* = 0.82) (Table 2).

#### 3.1.3 FSKT-mult

There was a significant effect of condition (Chi^2^ = 78.185; *p* < 0.001; *df* = 7; *N* = 16). The performance was lower in control condition compared to CA + FSSMPG (*z* = −3.52; *p* < 0.001; *r* = 0.88), CA + SSSMPG (*z* = −3.36; *p* = 0.001; *r* = 0.84), CA + FPSMPG (*z* = −3.36; *p* = 0.001; *r* = 0.84), CA + SPSMPG (*z* = −3.39; *p* = 0.001; *r* = 0.85), CA + FPSMNPG (*z* = −2.92; *p* = 0.003; *r* = 0.73) and CA + SPSMNPG (*z* = −2.02; *p* = 0.043; *r* = 0.51). In addition, values were lower in CA + NM condition compared to CA + FSSMPG (*z* = −3.52; *p* < 0.001; *r* = 0.88), CA + SSSMPG (*z* = −3.41; *p* = 0.001; *r* = 0.85), CA + FPSMPG (*z* = −3.53; *p* < 0.001; *r* = 0.88), CA + SPSMPG (*z* = −3.27; *p* = 0.001; *r* = 0.82) and CA + FPSMNPG (*z* = −2.11; *p* = 0.035; *r* = 0.53). Performance was higher in CA + FSSMPG condition compared to CA + SSSMPG (*z* = −3.52; *p* < 0.001; *r* = 0.88), and CA + FPSMPG (*z* = −3.52; *p* < 0.001; *r* = 0.88), CA + SPSMPG (*z* = −3.52; *p* < 0.001; *r* = 0.88), CA + FPSMPNG (*z* = −3.52; *p* < 0.001; *r* = 0.88), and CA + SPSMNPG (*z* = −3.52; *p* < 0.001; *r* = 0.88). Moreover, higher performance was recorded in CA + SSSMPG condition in comparison to CA + FPSMNPG (*z* = −2.06; *p* = 0.039; *r* = 0.52) and CA + SPSMNPG (*z* = −3.3; *p* = 0.001; *r* = 0.83). Values were higher in CA + FPSMPG condition as compared to CA + FPSMNPG (*z* = −2.54; *p* = 0.011; *r* = 0.64) and CA + SPSMNPG (*z* = −3.53; *p* < 0.001, *r* = 0.88) and higher in CA + SPSMPG condition as compared to CA + SPSMNPG (*z* = −3.18; *p* = 0.001; *r* = 0.80) and CA + FPSMNPG (*z* = −2.14; *p* = 0.032; *r* = 0.54) (Table 2).

### 3.2 Perceived exertion

#### 3.2.1 RPE_TSAT

There was a significant effect of condition (Chi^2^ = 76.93; *p* < 0.001; *df* = 7; *N* = 16), with higher scores in control condition compared to CA + FSSMPG (*z* = −3.60; *p* < 0.001; *r* = 0.9), CA + SSSMPG (*z* = −3.56; *p* < 0.001; *r* = 0.89), CA + FPSMPG (*z* = −3.02; p = 0.003; *r* = 0.76), CA + FPSMNPG (*z* = −3.25; *p* = 0.001; *r* = 0.81), CA + SPSMNPG (*z* = −2.8; *p* = 0.005; *r* = 0.70). Also, scores were higher in CA + NM condition compared to CA + FSSMPG (*z* = −3.56; *p* < 0.001; *r* = 0.89), CA + SSSMPG (*z* = −3.59; *p* < 0.001; *r* = 0.90), CA + FPSMPG (*z* = −3.27; *p* = 0.001; *r* = 0.82), CA + FPSMNPG (*z* = −2.71; *p* = 0.007; *r* = 0.68) and CA + SPSMPG (*z* = −2.99; *p* = 0.003; *r* = 0.75). The scores were lower in CA + FSSMPG condition as compared to CA + FPSMPG (*z* = −3.48; *p* = 0.001; *r* = 0.87), CA + SSSMPG (*z* = −3.56; *p* < 0.001; *r* = 0.89), CA + FPSMNPG (*z* = −2.99; *p* = 0.003; *r* = 0.75), CA + SPSMNPG (*z* = −3.33; *p* = 0.001; *r* = 0.83). Lower values were recorded in CA + SSSMPG condition compared to CA + FPSMPG (*z* = −2.78; *p* = 0.005; *r* = 0.70), CA + SPSMPG (*z* = −3.44; *p* = 0.001; *r* = 0.86), CA + FPSMNPG (*z* = −3.09; *p* = 0.002; *r* = 0.77) and CA + SPSMNPG (*z* = −2.81; *p* = 0.005; *r* = 0.70). The scores were lower in CA + FPSMPG condition compared to CA + SPSMPG (*z* = −3.24; *p* = 0.001; *r* = 0.81) and CA + SPSMNPG (*z* = −2.68; *p* = 0.007; *r* = 0.67) (Table 3).

**Table 3:** Effects of warm-up music and conditioning activity on perceived exertion (RPE) following taekwondo specific agility test (TSAT), frequency speed kick test 10s (FSKT 10s) and multiple frequency speed kick test (FSKT-mult).

Test	Control	CA + NM	CA + FSSMPG	CA + SSSMPG	CA + FPSMPG	CA + SPSMPG	CA + FPSMNPG	CA + SPSMNPG
RPE-TSAT: Med/IQR	4.5/1	4.5/2	1/1^a^^,^^c^^,^^f^^,^^d^^,^^i^^,^^m^	2/2^a^^,^^c^^,^^f^^,^^k^^,^^i^^,^^m^	3/1^b^^,^^β^^,^^k^^,^^m^	4/1^β^	3.5/2^b^^,^^β^	3/1.75^b^
RPE-FSKT-10s: Med/IQR	5/0.75	5/1	3/0.75^a^^,^^c^^,^^j^^,^^f^^,^^k^^,^^ɸ^	3.81/2^b^^,^^β^^,^^i^^,^^m^	4/1^b^^,^^β^^,^^m^	4/1^b^^,^^β^	5/1^β^	6/1
RPE-FSKT-mult: Med/IQR	9/0.75	9/1/	7/2^a^^,^^β^^,^^j^^,^^f^^,^^k^^,^^m^^,^^i^	8/2^b^^,^^β^	8/1^b^^,^^β^^,^^i^^,^*	8/2^b^^,^^β^^,^^m^	8/0^a^^,^^β^^,^^m^	8/1^b^^,^^β^

Med/IQR, values as Median/ Interquartile; CA + NM, Conditioning activity without music (NM); CA + FSSMPG, conditioning activity with fast (F), self-selected music (SSM) from preferred genre (PG), CA + SSSMPG, CA with slow (S), self-selected music (SSM) from preferred genre (PG); CA + FPSMPG, conditioning activity with fast (F), pre-selected music (PSM) from preferred genre (PG); CA + SPSMPG, conditioning activity with slow (S), pre-selected music (PSM) from preferred genre (PG); CA + FPSMNPG, conditioning activity with fast (F), pre-selected music (PSM) from non-preferred genre (NPG); CA + SPSMNPG, conditioning activity with slow (S) pre-selected music (PSM) from non-preferred genre (NPG).

^a^ Different from control at *p* < 0.001.

^b^ Different from control at *p* < 0.05.

^c^ Different from CA + NM at *p* < 0.001.

^β^ Different from CA + NM at *p* < 0.05.

^d^ Different from CA + SSSMPG at *p* < 0.001.

^j^ Different from CA + SSSMPG at *p* < 0.05.

^e^ Different from CA + FPSMPG at *p* < 0.001.

^f^ Different from CA + FPSMPG at *p* < 0.05.

^g^ Different from CA + SPSMPG at *p* < 0.001.

^k^ Different from CA + SPSMPG at *p* < 0.05.

^ɸ^ Different from CA + FPSMNPG at *p* < 0.001.

^i^ Different from CA + FPSMNPG at *p* < 0.05.

*Different from CA + SPSMNPG at *p* < 0.001.

^m^ Different from CA + SPSMNPG at *p* < 0.05.

#### 3.2.2 RPE_FSKT-10s

There was a significant effect of condition (Chi^2^ = 62.462; *p* < 0.001; *df* = 7; *N* = 16), with higher scores in control condition compared to CA + FSSMPG (*z* = −3.56; *p* < 0.001; *r* = 0.89), CA + SSSMPG (*z* = −2.77; *p* = 0.006; *r* = 0.69), CA + FPSMPG (*z* = −2.91; *p* = 0.004; *r* = 0.73), and CA + SPSMPG (*z* = −2.55; *p* = 0.011; *r* = 0.64). Also, values were higher in CA + NM condition compared to CA + FSSMPG (*z* = −3.59; *p* < 0.001; *r* = 0.90), CA + SSSMPG (*z* = −3.18; *p* = 0.001; *r* = 0.80), CA + FPSMPG (*z* = −3.27; *p* = 0.001; *r* = 0.82), CA + SPSMPG (*z* = −2.95; *p* = 0.003; *r* = 0.74) and CA + FPSMNPG (*z* = −2.67; *p* = 0.008; *r* = 0.67). RPE scores were lower in CA + FSSMPG condition compared to CA + SSSMPG (*z* = −2.05; *p* = 0.04; *r* = 0.51), CA + FPSMPG (*z* = −3; *p* = 0.003; *r* = 0.75), CA + SPSMPG (*z* = −3.09; *p* = 0.002; *r* = 0.77), CA + FPSMNPG (*z* = −3.48; *p* < 0.001; *r* = 0.87), and CA + SPSMNPG (*z* = −3.56; *p* < 0.001; *r* = 0.89). The scores were lower in CA + SSSMPG condition compared to CA + FPSMNPG (*z* = −2.55; *p* = 0.011; *r* = 0.64), CA + SPSMNPG (*z* = −3.1; *p* = 0.002; *r* = 0.78). The scores were lower in CA + FPSMPG condition compared to CA + SPSMNPG (*z* = −3.23; *p* = 0.001; *r* = 0.81) (Table 3).

#### 3.2.3 RPE_FSKT-mult

There was a significant effect of condition (Chi^2^ = 53.008; *p* < 0.001; *df* = 7; *N* = 16). The scores were higher in control condition as compared to CA + FSSMPG (*z* = −3.58; *p* < 0.001; *r* = 0.90), CA + SSSMPG (*z* = −3; *p* = 0.003; *r* = 0.75), CA + FPSMPG (*z* = −3.12; *p* = 0.002; *r* = 0.78), CA + SPSMPG (*z* = −2.99; *p* = 0.003; *r* = 0.75), CA + FPSMNPG (*z* = −3.57; *p* < 0.001; *r* = 0.89) and CA + SPSMNPG (*z* = −2.67; *p* = 0.008; *r* = 0.67). Also, scores were higher in CA + NM condition compared to CA + FSSMPG (*z* = −3.46; *p* = 0.001; *r* = 0.87), CA + SSSMPG (*z* = −2.57; *p* = 0.01; *r* = 0.64), CA + FPSMPG (*z* = −3.55; *p* = 0.011; *r* = 0.89), CA + SPSMPG (*z* = −2.65; *p* = 0.008; *r* = 0.66), CA + FPSMNPG (*z* = −2.68; *p* = 0.007; *r* = 0.67) and CA + SPSMNPG (*z* = −1.99; *p* = 0.046; *r* = 0.50). In addition, lower scores were recorded in CA + FSSMPG condition compared to CA + SSSMPG (*z* = −2.87; *p* = 0.004; *r* = 0.72), CA + FPSMPG (*z* = −2.37; *p* = 0.018; *r* = 0.59), CA + SPSMPG (*z* = −2.43; *p* = 0.015; *r* = 0.61), CA + FPSMNPG (*z* = −3.22; *p* = 0.001; *r* = 0.81), CA + SPSMNPG (*z* = −3.26; *p* = 0.001; *r* = 0.82) and higher in CA + FPSMPG condition compared to CA + FPSMNPG (*z* = −2.54; *p* = 0.011; *r* = 0.64) and CA + SPSMNPG (*z* = −3.53; *p* < 0.001; *r* = 0.88). The scores were lower in the CA + SPSMPG condition as compared to CA + SPSMNPG (*z* = −2.31; *p* = 0.021; *r* = 0.58). The scores were lower in the CA + FPSMNPG as compared to CA + SPSMNPG (*z* = −2.11; *p* = 0.035; *r* = 0.53) (Table 3).

### 3.3 Psychological responses

#### 3.3.1 Felt arousal scale

There was a significant effect of condition (Chi^2^ = 95.079; *p* < 0.001; *df* = 7; *N* = 16). The scores were lower in control condition compared to CA + NM (*z* = −2.31; *p* = 0.021; *r* = 0.58), CA + FSSMPG (*z* = −3.64; *p* < 0.001. *r* = 0.91), CA + SSSMPG (*z* = −3.61; *p* < 0.001; *r* = 0.90), CA + FPSMPG (*z* = −3.58; *p* < 0.001; *r* = 0.90), CA + SPSMPG (*z* = −3.45; *p* = 0.001; *r* = 0.86) and CA + FPSMNPG (*z* = −3.01; *p* = 0.011; *r* = 0.75). Also, CA + NM condition resulted in lower scores compared to CA + FSSMPG (*z* = −3.55; *p* < 0.001; *r* = 0.89), CA + SSSMPG (*z* = −3.62; *p* < 0.001; *r* = 0.91), CA + FPSMPG (*z* = −3.45; *p* = 0.001; *r* = 0.86), CA + SPSMPG (*z* = −3.25; *p* = 0.001; *r* = 0.81) and CA + FPSMNPG (*z* = −2.95; *p* = 0.003; *r* = 0.74). Higher scores were recorded in CA + FSSMPG condition in comparison to CA + SSSMPG (*z* = −3.58; *p* < 0.001; *r* = 0.90), CA + FPSMPG (*z* = −3.56; *p* < 0.001; *r* = 0.89), CA + SPSMPG (*z* = −3.55; *p* < 0.001; *r* = 0.89), CA + FPSMNPG (*z* = −3.54; *p* < 0.001; *r* = 0.89) and CA + SPSMNPG (*z* = −3.57; *p* < 0.001; *r* = 0.89). Moreover, FAS values were higher in CA + SSSMPG compared to CA + SPSMPG (*z* = −2.65; *p* = 0.008; *r* = 0.66), CA + FPSMNPG (*z* = −3.14; *p* = 0.002; *r* = 0.79) and CA + SSSMNPG (*z* = −3.61; *p* < 0.001; *r* = 0.90). The scores were higher in CA + FPSMPG condition as compared to CA + FPSMNPG (*z* = −2.39; *p* = 0.017; *r* = 0.60) and CA + SPSMNPG (*z* = −3.58; *p* < 0.001; *r* = 0.90). In addition, CA + SPSMPG condition resulted in higher scores compared to CA + FPSMNPG (*z* = −2.11; *p* = 0.035; *r* = 0.53) and CA + SPSMNPG (*z* = −3.34; *p* = 0.001; *r* = 0.84) and CA + FPSMNPG elicited higher scores as compared to CA + SPSMNPG (*z* = −2.98; *p* = 0.003; *r* = 0.75) (Table 4).

**Table 4:** Effects of warm-up music and conditioning activity on motivation rate, feeling scale (FS) and felt arousal scale (FAS).

Test	Control	CA + NM	CA + FSSMPG	CA + SSSMPG	CA + FPSMPG	CA + SPSMPG	CA + FPSMNPG	CA + SPSMNPG
Motivation: Med/IQR	2/1	2/0.75	8/0.75^a^^,^^c^^,^^d^^,^^e^^,^^g^^,^^ɸ^^,^*	6/0^a^^,^^c^^,^^f^^,^^k^^,^^i^^,^*	5/1.5^a^^,^^c^^,^*,^i^^,^^k^	4/1.75^b^^,^^β^^,^^m^	4/2.75^b^^,^^β^^,^^m^	1/1^β^
FS: Med/IQR	−1/3	0/0	4/1^a^^,^^β^^,^^d^^,^^f^^,^^k^^,^^ɸ^^,^*	1/1^b^^,^^β^^,^^f^^,^^i^^,^^m^	2/0^b^^,^^c^^,^^k^^,^^ɸ^	1.5/2.75^b^^,^^β^^,^^i^^,^^m^	−1/1.75^β^	−1/2
FAS: Med/IQR	1/0	2/1^b^	6/1^a^^,^^c^^,^^d^^,^^e^^,^^g^^,^^ɸ^^,^*	4/1^a^^,^^c^^,^^k^^,^^i^^,^*	4/1^a^^,^^β^^,^^i^^,^*	3/2^b^^,^^β^^,^^i^^,^^m^	3/1^b^^,^^β^^,^^m^	1/1

Med/IQR, values as Median/ Interquartile; CA + NM, Conditioning activity without music (NM); CA + FSSMPG, conditioning activity with fast (F), self-selected music (SSM) from preferred genre (PG), CA + SSSMPG, CA with slow (S), self-selected music (SSM) from preferred genre (PG); CA + FPSMPG, conditioning activity with fast (F), pre-selected music (PSM) from preferred genre (PG); CA + SPSMPG, conditioning activity with slow (S), pre-selected music (PSM) from preferred genre (PG); CA + FPSMNPG, conditioning activity with fast (F), pre-selected music (PSM) from non-preferred genre (NPG); CA + SPSMNPG, conditioning activity with slow (S) pre-selected music (PSM) from non-preferred genre (NPG).

^a^ Different from control at *p* < 0.001.

^b^ Different from control at *p* < 0.05.

^c^ Different from CA + NM at *p* < 0.001.

^β^ Different from CA + NM at *p* < 0.05.

^d^ Different from CA + SSSMPG at *p* < 0.001.

^j^ Different from CA + SSSMPG at *p* < 0.05.

^e^ Different from CA + FPSMPG at *p* < 0.001.

^f^ Different from CA + FPSMPG at *p* < 0.05.

^g^ Different from CA + SPSMPG at *p* < 0.001.

^k^ Different from CA + SPSMPG at *p* < 0.05.

^ɸ^ Different from CA + FPSMNPG at *p* < 0.001.

^i^ Different from CA + FPSMNPG at *p* < 0.05.

*Different from CA + SPSMNPG at *p* < 0.001.

^m^ Different from CA + SPSMNPG at *p* < 0.05.

#### 3.3.2 Feeling scale

There was a significant effect of condition (Chi^2^ = 79.540; *p* < 0.001; *df* = 7; *N* = 16), with values were lower in the control condition compared to CA + FSSMPG (*z* = −3.55; *p* < 0.001; *r* = 0.89), CA + SSSMPG (*z* = −2.97; *p* = 0.003; *r* = 0.74), CA + FPSMPG (*z* = −3.44; *p* = 0.001; *r* = 0.86) and CA + SPSMPG (*z* = −2.54; *p* = 0.011; *r* = 0.64). Moreover, scores were lower in CA + NM condition compared to CA + FSSMPG (*z* = −2.33; *p* = 0.02; *r* = 0.58), CA + SSSMPG (*z* = −3.11; *p* = 0.002; *r* = 0.78), CA + FPSMPG (*z* = −3.56; *p* < 0.001; *r* = 0.89), CA + SPSMPG (*z* = −2.08; *p* = 0.038; *r* = 0.52), CA + FPSMNPG (*z* = −2.33; *p* = 0.02; *r* = 0.58). In addition, higher scores were recorded in CA + FSSMPG condition compared to CA + SSSMPG (*z* = −3.56; *p* < 0.001; *r* = 0.89), CA + FPSMPG (*z* = −3.36; *p* = 0.001; *r* = 0.84), CA + SPSMPG (*z* = −3.43; *p* = 0.001; *r* = 0.86), CA + FPSMNPG (*z* = −3.53; *p* < 0.001; *r* = 0.88) and CA + SPSMNPG (*z* = −3.53; *p* < 0.001; *r* = 0.88). Furthermore, CA + SSSMPG resulted in higher scores compared to CA + FPSMPG (*z* = −3.05; *p* = 0.002; *r* = 0.76), CA + FPSMNPG (*z* = −3.32; *p* = 0.001; *r* = 0.83) and CA + SPSMNPG (*z* = −3.19; *p* = 0.001; *r* = 0.80) and higher scores were recorded in CA + FPSMPG condition in comparison to CA + SPSMPG (*z* = −2.34; *p* = 0.019; *r* = 0.59), and CA + FPSMNPG (*z* = −3.56; *p* < 0.001; *r* = 0.89). Scores were higher in CA + SPSMPG condition compared to CA + FPSMNPG (*z* = −3.17; *p* = 0.002; *r* = 0.79) and CA + SPSMNPG (*z* = −3.09; *p* = 0.002; *r* = 0.77).

#### 3.3.3 Motivation

There was a significant effect of condition (Chi^2^ = 97.881; *p* < 0.001; *df* = 7). Control condition induced lower scores compared to CA + FSSMPG (*z* = −3.58; *p* < 0.001; *r* = 0.90), CA + SSSMPG (*z* = −3.57; *p* < 0.001; *r* = 0.89), CA + FPSMPG (*z* = −3.57; *p* < 0.001; *r* = 0.89), CA + SPSMPG (*z* = −3.34; *p* = 0.001; *r* = 0.84) and CA + FPSMNPG (*z* = −3.11; *p* = 0.002; *r* = 0.87). Moreover, lower scores were recorded under CA + NM condition compared to CA + FSSMPG (*z* = −3.56; *p* < 0.001; *r* = 0.89), CA + SSSMPG (*z* = −3.57; *p* < 0.001; *r* = 0.89), CA + FPSMPG (*z* = −3.56; *p* < 0.001; *r* = 0.89), CA + SPSMPG (*z* = −2.72; *p* = 0.007; *r* = 0.68), CA + FPSMNPG (*z* = −2.28; *p* = 0.022; *r* = 0.57) and CA + SPSMNPG (*z* = −3.35; *p* = 0.001; *r* = 0.84). In addition, scores were higher in CA + FSSMPG condition in comparison to CA + SSSMPG (*z* = −3.58; *p* < 0.001; *r* = 0.90), CA + FPSMPG (*z* = −3.56; *p* < 0.001; *r* = 0.89), CA + SPSMPG (*z* = −3.54; *p* < 0.001; *r* = 0.90), CA + FPSMNPG (*z* = −3.56; *p* < 0.001; *r* = 0.89) and CA + SPSMNPG (*z* = −3.56; *p* < 0.001; *r* = 0.89). The scores were higher in CA + SSSMPG as compared to CA + FPSMPG (*z* = −2.96; *p* = 0.003; *r* = 0.74), CA + SPSMPG (*z* = −3.32; *p* = 0.001; *r* = 0.83), CA + FPSMNPG (*z* = −3.31; *p* = 0.001; *r* = 0.83) and CA + SPSMNPG (*z* = −3.57; *p* < 0.001; *r* = 0.89) and also higher in CA + FPSMPG condition compared to CA + SPSMPG (*z* = −2.58; *p* = 0.01; *r* = 0.65), CA + FPSMNPG (*z* = −2.57; *p* = 0.01; *r* = 0.64) and CA + SPSMNPG (*z* = −3.57; *p* < 0.001; *r* = 0.89). Additionally, scores were higher in CA + SPSMPG condition compared to CA + SPSMNPG (*z* = −3.43; *p* = 0.001; *r* = 0.86) and higher in CA + FPSMNPG in comparison to CA + SPSMNPG (*z* = −3.32; *p* = 0.001; *r* = 0.83) (Table 4).

## 4 Discussion

While music and PAPE have been studied independently in the context of exercise, current findings are among the first to assess the combined effects of CA intervention and listening to music during warm-up with varied selection process and intrinsic components (i.e., tempos, and genre) on the subsequent TKD specific performances and psychological-related responses. Findings from the present study revealed that combining a plyometric-based CA with a self-selected music at fast or slow tempos improved TSAT, FSK-10s, FSKT-mult, FAS, motivation and RPE post-TSAT better than the control, CA single use and the combined CA and pre-selected (i.e., fast and slow) music conditions. Moreover, combining a plyometric-based CA and fast self-selected music induced greater FS, RPE-FSKT-10s and RPE-FSKT-mult values than control, CA single use, the combined CA and slow self-selected music and the combined CA and pre-selected (i.e., fast and slow) music conditions.

The effectiveness of including a plyometric CA within warm-up to induce PAPE in TKD specific performances has been previously documented (9, 10, 15), but results were not all consistent (11, 13). Similarly, warm-up music was an effective ergogenic aid in some circumstances (9, 15, 36–38), while no effectiveness has been reported in other studies (39, 40). In the present study, combining self-selected music with a plyometric CA resulted in greater performance than with CA single use as well with the CA and pre-selected music combinations. The two interventions are fundamentally different with music being classified as a psychological ergogenic aid (18) and CA as a physical stimulus (6). Therefore, the reported results generated by their combination are not surprising, since the physical abilities and psychological aspects are interrelated and affect each other (27). In fact, under preferred conditions, warm-up music may increase the anticipatory response to exercise (25), resulting in increased effort and muscular force development (19, 20). In this consideration, listening to music during a warm-up session has been shown to increase plasma catecholamine concentrations, which may promote muscular activation, shift blood flow toward skeletal muscles, and modify metabolic reactions during afterward exercise (41). From a PAPE perspective, it is well-documented that performance enhancement is the result of an improvement of muscular activation and neural drive (6, 7). Plyometric exercises are particularly well known to feature quicker shortening-stretching cycles and boost movement speed (12). Therefore, the larger performance improvement observed under both fast and slow self-selected music conditions may be attributed to a PAPE window of action that is prolonged as a result of synergistic effects at the central (e.g., neural drive, hormonal secretion) (12, 18) and peripheral levels (e.g., power production and motors units synchronization) (6, 41).

It has been demonstrated that brain activity changes generated by listening to music are highly reliant on the individual's musical selection, with a stronger emotional attachment to preferred music possibly altering responses (42). Especially, performance enhancements are mediated by music's ability to increase motivation to exercise and regulate arousal (43, 44). In the present study, affective valence (i.e., FAS and FS) and motivation were higher when CA was combined with fast or slow self-selected music. The recorded results may implicate a synergetic effect by changes in arousal level since positive arousal increases are associated with improved muscular force output (45). According to Blazevich and Babault (6), the level of familiarity with exercise and motivation could have detrimental effects on PAPE stimulus. In the present study, the recruited athletes were well-familiarized with the specific tasks. Regarding motivation, results from combining CA with SSMPG could be supported by previous findings reporting increased motivation and intent to give effort with preferred music (46, 47). Such effects of increased motivation may last throughout the exercise bout, even if the selected music was played only during the warm-up (38).

Music has been reported to reduce perceived exertion due to dissociative effects from physical exercise cues (18). However, the potential of warm-up music on the dissociation of unpleasant effects from exercise declined over time (36), even when music was self-selected (38). In the present study, RPE decreased under the CA combined with fast and slow music self-selected within preferred genre. This result could indicate that listening to music under preferred condition while performing a CA extends its effect on fatigue perception. This could be related to a generated PAPE from the combined stimulus resulting in lower muscular fatigue (6, 41). PAPE could emerge from the reduction in the inhibitory effects of sensory afferents (through reduced pre-synaptic inhibition) or an increase in motoneuron excitability (6). However, integrating a CA within athletes' warm-up sessions should be aware of the fatigue-potentiation relationship and the appropriate timing to achieve maximal performance (48). In this consideration, it is understood that a PAPE stimulus requires a sufficient rest period before performing a subsequent movement (4). In the present study, three minutes of rest were given before performing the tests were an adequate rest interval. This could be evident as using a plyometric CA may cause low exhaustion, enabling a strong potentiation effect while shortening the time required for the optimal PAPE benefit (5). In this consideration, Ouergui et al. (15) reported that when using plyometric activity greater performance enhancement was documented with 3 min than with 7 min regardless of the effort/pause ratio. From other side, the level of PAPE has been reported to be greater in trained individuals since they have been shown to display faster recovery than untrained ones (14). Nuzzo et al. (49) found that the increased excitability was detectable immediately after the bout of contractions and slowly decreased to baseline, but not before at least 20 min. In the present study, the fact that CA and SSMPG greater effects persisted over consecutive tasks could indicate that short-duration contractions would not allow fatigue increase during contractions, while still promoting PAPE (48). This could be evident since the given rest interval between tests would allow muscle recovery, enhancing the prevalence of PAPE over fatigue (50). PAPE may occur as a result of the alteration in the stimulation pattern of motor units, increasing the number of excited motor units (51). This effect of activation history on the subsequent force production might be greater when subsequent exercise simulates the previously activated muscle fibers (52). This could be attributed to a better synchronization of the motor units involved, and a reduction in presynaptic inhibition (3).

The current study could have important implications in the field of combat sports, especially, in TKD, and serve as a practical framework to improve athletes' performances. Specifically, based on the difference between the two stimuli in terms of their nature (i.e., physical vs. psychological), combining music and CA during warm-up session might be a useful tool to enhance physical fitness and mental performance. In competitions scenarios, these two pre-event procedures could reduce the anticipated stress (i.e., improving affective state and motivation) while enhancing power production (i.e., PAPE phenomenon). In training settings, starting sessions with music and plyometric-based CA may help to attain objectives with lower time and energy costs. However, findings should be taken with caution and some limitations should be considered in future investigations. In fact, the results of the present study are conducted only within male athletes and thus cannot be generalized to female practitioners. Moreover, the physiological mechanisms behind the performance enhancement were not measured. Furthermore, using simulated combats could modulate the usefulness of combining warm-up music with a CA through increasing psychological stress and fatigue level.

## 5 Conclusions

Listening to fast self-selected warm-up music from preferred genre in combination with a plyometric CA is an effective strategy to improve specific performances and the associated psychological responses of TKD athletes. Moreover, accompanying a plyometric CA with self-selected music from preferred genre could improve exercise intensity while reducing the perceived exertion. Taking into consideration that benefits, from the two stimuli combination, were greater under self-selected conditions, providing listeners with the opportunity to select their preferred music without restrictions is recommended. Besides, the present study emphasizes the need to individualize warm-up strategies according to each athlete personal effects.

## Data Availability

The raw data supporting the conclusions of this article will be made available by the authors, without undue reservation.
